# Underlying Kidney Diseases and Complications for COVID-19: A Review

**DOI:** 10.3389/fmed.2020.600144

**Published:** 2020-11-23

**Authors:** Prince Dadson, Comfort Dede Tetteh, Eleni Rebelos, Robert M. Badeau, Dariusz Moczulski

**Affiliations:** ^1^Department of Internal Medicine and Nephrodiabetology, Medical University of Łódz, Łódz, Poland; ^2^Department of Epidemiology and Biostatistics, University of the Witwatersrand, Johannesburg, South Africa; ^3^Turku PET Centre, University of Turku, Turku, Finland; ^4^Department of Health Science, Indiana Institute of Technology, Fort Wayne, IN, United States

**Keywords:** SARS-CoV-2, COVID-19, kidney disease, kidney transplantation, acute kidney injury

## Abstract

There is mounting evidence supporting that patients with kidney diseases are particularly vulnerable to coronavirus disease-2019 (COVID-19) caused by the severe acute respiratory syndrome coronavirus-2 (SARS-CoV-2). The review was conducted to examine the risk and complications of COVID-19 among patients with confirmed cases of underlying kidney disease. A search of Google Scholar, PubMed and Science direct databases to August 2020 was conducted using search terms pertaining to kidney diseases, renal insufficiency, kidney injury, angiotensin receptors, hemodialysis, and kidney transplant. We briefly reviewed COVID-19 in the context of kidney diseases. A significant proportion of hospitalized patients for COVID-19 have acute kidney injury, which further deteriorates their prognosis. COVID-19 increases morbidity and mortality among people already diagnosed with kidney disorders and obesity due to multiple organ injury caused by the SARS-CoV-2. This review supports the need for clinicians to carefully manage and monitor all patients with renal disorders in order to minimize acute kidney injuries. Although some therapeutic drugs have been suggested by some studies, treatment should be administered cautiously not to worsen the condition of the kidney. Further studies are required to highlight the efficient management of patients with underlying kidney diseases, who are infected with SARS-CoV-2. With proactive systematic screening and triaging, close monitoring and prompt management of coexisting other infections, the COVID-19 disease burden among these patients could be reduced.

## Introduction

In late December 2019, cases of pneumonia of unknown origin were identified in Wuhan, the capital city of Hubei province in central China. The causative pathogen has since been identified as a severe acute respiratory syndrome coronavirus-(2) (SARS-CoV-2) due to the phylogenetic similarity to the previously isolated SARS-CoV (1–3).

The coronavirus disease 2019 (COVID-19) caused by SARS-CoV-2 has been declared as a pandemic and has infected ~21.2 million people that caused over 761,000 deaths across countries and territories globally as at August 2020 (4, 5). These exponential numbers suggest that COVID-19 is a threat to humanity and a major public health problem (2). COVID-19 presents as an acute upper and lower respiratory tract infection leading to pneumonia and also affects multiple other tissues and organs including the kidneys (6). Some documented common clinical signs and symptoms associated with the disease include fever, cough, difficulty in breathing, fatigue, sore throat, and lung infection among others (7, 8).

Co-morbidities are linked to severe and critical cases of the COVID-19, with hypertension, diabetes mellitus, and cardiovascular diseases being the main causes (7). Studies have reported that patients with chronic underlying clinical conditions face the severest risk and complications due to COVID-19 (8–10). According to Chen et al. (7), COVID-19 can result in multiple organ failure, thus, it increases the risk of mortality especially among patients with chronic conditions. Most patients at the intensive care unit (ICU) are confirmed cases, who are reported to have co-morbidities. Recently, many studies have examined the impact of the COVID-19 on kidney diseases and the outcome of patients (11). Cheng et al. (12) found that 2% of confirmed cases in a prospective study have chronic kidney disease. Patients with a kidney transplant are also impacted alike with SARS-CoV-2 due to their immune-suppression status (13). Also, acute kidney injury (AKI) is reported to be a common risk factor precipitating severity and fatality among patients (7, 8, 12, 14, 15).

This review aims to examine risk and complications of COVID-19 among confirmed cases with underlying kidney conditions to guide clinical practice and management decisions.

## Methodology

A search was conducted on studies published from first January to tenth June 2020 in Google Scholar, PubMed, and Science Direct databases. Search terms such as “COVID-19,” “SARS-CoV-2,” “SARS2,” “kidney diseases,” “renal insufficiency,” “kidney injury,” angiotensin receptors,” “hemodialysis,” and “kidney transplant” were used with no language restriction introduced in the search. The reference list of each selected study was checked to ensure that no relevant study was missed. Eligible studies were those that described and reported on the risk, complications, and management of COVID-19 disease among people with underlying kidney diseases. Additionally, some unpublished reports found online were also included. Studies excluded from the review were those that reported on other co-morbidities but not kidney diseases as well as duplicated studies and news articles. A total of 260 published articles were reviewed, but only 87 were included after a careful review.

## SARS-CoV-2 Infection and Kidney Diseases

### The Renin-Angiotensin-Aldosterone System (RAAS)

The RAAS plays a cardinal role in human renal and cardiovascular physiology and pathophysiology. Briefly, angiotensinogen released by the liver, is transformed to angiotensin I by the action of renin. Angiotensin I is converted to angiotensin II (AII) through removal of two C-terminal residues by the angiotensin-converting enzyme (ACE). Besides the well-described role of ACE, which is homologous to ACE protease, ACE2 has also been discovered. ACE2 is mainly located in the membrane cell, or found as circulating forms, and it degrades AII to Ang (1–7), and therefore ACE2 acts a functional clearance mechanism for AII. Ang (1–7) is also an active peptide that antagonizes the actions of AII, through a G-protein coupled receptor. Importantly, ACE2 acts as a receptor for SARS-CoV-2 (16), and thus in the presence of SARS-CoV2 infection, AII metabolism is attenuated, thus leading to pulmonary vasoconstriction and inflammation. Even though the receptor action of ACE2 located in the cell membranes, promotes entry of the SARS-CoV-2, the circulating soluble ACE2 enzyme, may offer a therapeutic possibility, since it binds to the virus, decreasing its availability to the membrane ACE2 receptors and thus minimizing cell infection (17).

### Epidemiology and Clinical Manifestation of SARS-CoV-2 in Renal Diseases

Since the emergence of COVID-19 in December 2019, it has attained a worldwide distribution (9, 10, 18). Although most of the early cases had a direct linkage to the seafood market in Wuhan city (2), recent cases are a result of community or human-to-human transmission (19, 20). According to Li et al. (2), the average incubation period of the disease is 5.2 days (95% Confidence Interval: 4.1–7.0).

Clinical manifestations include cough, shortness of breath, muscle ache, confusion, headache, sore throat, rhinorrhea, and chest pain among others (7, 8). A systematic review and meta-analysis indicate a 1% (95% CI, 1–2%) pooled prevalence of chronic kidney diseases with about 83.9% suffering a severe COVID-19 that resulted in a 53% mortality rate (21). Chronic kidney disease (CKD) is one of the common underlying diseases among COVID-19 hospitalized patients (22).

### The Mechanism of Action of SARS-CoV-2 in the Kidney

Direct cellular injury due to the virus or sepsis leading to a cytokine storm syndrome are thought to be possible mechanisms connected to the SARS-CoV-2 disease. Recently, some studies show that the kidney is one of the target organs for SARS-CoV-2. The vulnerability of patients with diagnosed kidney conditions to SARS-CoV-2 is now known with its associated complications (11–13, 15, 21, 23). The vulnerability of these patients includes comorbidities of diabetes and hypertension, and immunosuppressive therapies among others that impair their immune system and cause their kidneys to fail (9, 11, 24).

### Pathogenesis of SARS-CoV-2 and Acute Kidney Injury and Mechanisms Underlying Worse Outcomes

Kidney impairment has been found to occur in some COVID-19 patients. About >50 % of patients admitted at the ICU present with acute kidney injury (AKI) characterized by elevated serum creatinine, reduced urine output, or both as a result of multiple factors (25). Earlier reports revealed incidence and mortality rates of 5–15 and 60–90%, respectively, for AKI cases (7, 12, 14). According to Li et al. (15), a significant number out of 193 hospitalized patients presented some form of kidney malfunction such as proteinuria (59%), hematuria (44%), elevated blood urea nitrogen (14%), and increased levels of creatinine (10%). Consistent with these findings by Li et al., Cheng et al. (12) found that about 3.2% of COVID-19 patients developed AKI at the initial stages of COVID-19 infection. Contrary to these findings, Wang et al. (10) reported that “COVID-19 disease does not result in AKI or aggravate Chronic Kidney Disease (CKD) among patients.”

Another retrospective study shows that SARS-CoV-2 directly affects the kidney tubules resulting in acute tubular damage and cytotoxicity, and thus, acute renal failure in COVID-19 patients especially among the aged and co-morbid (26). Additionally, early renal injury was significantly associated with C-reactive protein (CRP) and neutrophil ratio (NER) indicating that early renal injury was precipitated by severe infection (27). This study again revealed anomalies in the estimated glomerular filtration Rate (eGFR) of 66.7%, creatinine clearance (Ccr) of 41.7%, and elevated microalbuminuria (UACR) of 41.7%. These are all important biomarkers that can signify kidney impairment among severe COVID-19 patients and a poor outcome. They concluded that these measurements aided in the early detection of renal injury (27).

In a postmortem study on 26 patients who died from SARS-CoV-2 in China, by using ultrastructural and immunostaining assessment, “diffused acute proximal tubular injury with loss of brush border and non-isometric vacuolation” was found. This depicts a direct virulent nature of the novel virus (28). The authors again revealed that SARS-CoV-2 has a direct effect on the renal tubular epithelium and podocytes as associated with proteinuria and AKI in their study by using an indirect immunofluorescent (IF) method. This finding is in agreement with what Larsen et al. (24) found in a case report.

Thus, several mechanisms have been described through which SARS-CoV-2 can lead to acute and chronic kidney damage, such as via acute tubular necrosis, interstitial inflammation, injuries in renal vasculature and collapsing glomerulopathy. Endothelial dysfunction is a key characteristic of CKD *per se* already at an early stage (29) and endothelial damage such as pericyte detachment, expansion of sub-endothelial space and foam cell accumulation have been described in COVID-19 (30). Of note, ACE2 receptors are also expressed by endothelial cells (31), and the systemic endotheliitis in the course of COVID-19 can occur either by direct viral infection of the endothelium or it can be immune-mediated (32). Endothelial dysfunction leads to vasoconstriction-induced ischemia, inflammation and a procoagulant state. Hypoxia may also further aggravate renal damage (33), since the kidney is a richly perfused organ, receiving ~20% of cardiac output (34). Kidney injury may in turn further aggravate the clinical condition of patients with COVID-19, increasing the risk of pulmonary edema, thromboembolism and bleeding disorders (33). Indeed Cheng and colleagues showed that AKI during hospitalization in patients with COVID-19 lead to higher in-hospital mortality (12). As the authors discussed, patients with CKD who also develop COVID-19, and AKI have an already dysfunctional innate and adaptive immune system, which may also contribute to this worse prognosis (12).

### SARS-CoV-2 Among Kidney Transplant Patients

Viruses are the main cause of opportunistic infections post-transplant among kidney transplant recipients leading to increased morbidity and mortality (35). Factors including graft rejection, immune suppression, and tissue injury increase the chance of developing viral infection after kidney transplant (35). In the presence of pandemic, a case report found that kidney transplant patients present similar symptoms to what is portrayed in the general population (36). However, other clinical manifestations may include “hypoxia, chest crepitation, lymphopenia and high C-reactive protein.” Also, very high D dimer, ferritin, and troponin levels occurred among severe COVID-19 cases (13). Contrary to this, Guillen et al. (37), in a case report, disclosed atypical clinical symptoms such as viral gastroenteritis and oral dehydration, which calls on all clinicians to be aware of this vulnerable and at-risk population. Despite the risk among this population, a case report has documented a successful recovery of a kidney transplant patient infected with COVID-19. The treatment regimen used was temporary discontinuation of immunosupressants, and gradual reinstatement, and a low-dose methlprednisolone-based therapy (38). In agreement with this finding, another study showed that kidney transplant recipients infected with COVID-19 disease experienced a mild infection which can be managed based on their presenting symptoms (39). Lubetzky et al. (36) suggest that with a proactive systematic screening and triaging, close monitoring, and prompt management of coexisting other infections, COVID-19 disease burden among these patients will be minimal.

### Impact of SARS-CoV-2 on Dialysis Patients

The susceptibility of end-stage kidney failure patients on dialysis to COVID-19 disease due to their immune suppressed state is high (8, 40, 41). Some factors contributing to this high risk of contracting SARS-CoV-2 among hemodialysis (HD) patients and resulting in worse outcomes are multifactorial including: underlying comorbidities, aging, and immune suppression (41, 42). Additionally, their scheduled visits (i.e., thrice, weekly) to hemodialysis centers expose them to the outside increasing their risk of contracting COVID-19. It has been reported that, in spite of the hemodialysis patients being a high risk group, they are unlikely to progress to severe COVID-19 disease, although their immune function is impaired, and they are therefore unable to build up cytokines storms (43). In actuality, Huang et al. (8), have revealed that the processes involved leading to severe COVID-19 and increased mortality are due to an overresponse by the immune system with the cytokine storm against SARS-CoV-2. These findings suggest that this requires strict adherence and observation of Infection Prevention and Control (IPC) in hemodialysis centers and the management of hemodialytic patients using guidelines and standard operation procedures developed by the Nephrology Societies, Centers for Disease Control and Prevention and by the WHO. Evidently, Naicker et al. (6) have revealed that the hemodialysis centers remain a high risk area for SARS-CoV-2 transmission to hemodialysis patients, the families and healthcare workers alike.

### Therapeutic Management of Renal Disorder Patients With COVID-19

The cornerstone of successful treatment in this context is early detection of renal abnormalities, and avoidance of nephrotoxic agents. Further, it is essential to adhere to the recommended treatment guidelines to manage COVID-19 patients with kidney disorders. Drug management has aimed at targeting ACE2 in humans, which serves as a receptor for the virus (16). However, administration of glucocorticoids prevents the strong immune response and slows the cytokine storm especially among hemodialysis patients (7, 16). Also, the recommended therapy for kidney transplant recipients infected with COVID-19 is a low dose of methylprednisolone-based therapy (38). In all instances, the needed supportive care should be provided for patients with multiple organ failure. Further studies are required to show the success rate of all of these treatments.

## COVID-19 and Obesity

Obesity is an independent risk factor for development of CKD (44). The incidence and prevalence of obesity have reached epidemic dimensions and it is a public health challenge that affects high income, middle income and low income countries (45). A recent study by Popkin et al. (46), found that there is a 46% chance of obese people to be infected with SARS-CoV-2. Also, they are about 113% at risk of being hospitalized, 74% chance of being admitted at the ICU and are at 48% likely to die (47). This is because people with obesity present with many risk factors for COVID-19 such as hypertension, type 2 diabetes, dyslipidemia, chronic kidney or liver disease. A greater proportion of the total body fat is made up of white adipose tissue (WAT) which produces adipokines, metabolic active factors which play a vital role in the pathogenesis of obesity (48, 49). Angiotensinogen, leptin and cytokines are examples of adipokines (50). Expression of ACE2 in the adipose tissue is higher than in the lungs, making the adipose tissue more susceptible to SARS-CoV-2 which directly binds with the ACE2 on cell surfaces (51, 52), suggesting that the expanded adipose tissue in obese individuals is a potential viral reservoir, which leads to prolonged viral clearance. In line with this, previous reports have shown longer in-hospital stay and time for obtaining a negative swab in obese individuals (53, 54). Interestingly, in a study on female hypertensive patient's serum ACE2 levels correlated negatively with BMI (55) suggesting that decreased circulating ACE2 levels may contribute to the worse clinical picture in obese patients with COVID-19. Obese subjects have worse COVID-19 outcomes, including respiratory failure and increased mortality (52).

## Vitamin d and SARS-CoV-2

D hypovitaminosis is a common characteristic of patients with renal insufficiency and is associated with over activation of RAAS (56). Previous studies have shown an independent association between low serum concentration of 25-hydroxyl-vitamin D and susceptibility to acute respiratory tract infections (57). Ilie et al. reported negative correlations between the mean Vitamin D levels in various European countries with COVID-19 cases per million of population, and with COVID-19 mortality (58). Several clinical trials are currently investigating whether supplementation of Vitamin D can have beneficial effects on subjects with COVID-19.

In conclusion, this review revealed the current studies on patients with underlying kidney diseases infected with SARS-CoV-2. The COVID-19 increases morbidity and mortality among people already with kidney disorders due to multiple organ injury caused by the virus. There is a call on all clinicians to carefully manage and monitor all patients with renal disorders to minimize acute kidney injuries. Although some therapeutic drugs have been suggested by some studies, there is a need to tread cautiously not to worsen the condition of the kidney. Further studies are required to bring new information about the efficient management of patients with underlying kidney diseases infected with SARS-CoV-2.

## Author Contributions

PD: conceptualization. PD and CT: methodology, investigation, and writing—original draft. PD, ER, DM, and RB: writing—review and editing. PD and DM: supervision and project administration. All authors contributed to the article and approved the submitted version.

## Conflict of Interest

The authors declare that the research was conducted in the absence of any commercial or financial relationships that could be construed as a potential conflict of interest.
